# Impact of lockdown during the COVID-19 pandemic on number of patients and patterns of injuries at a level I trauma center

**DOI:** 10.1007/s00508-021-01824-z

**Published:** 2021-03-03

**Authors:** Arastoo Nia, Domenik Popp, Cornelia Diendorfer, Sebastian Apprich, Alexandru Munteanu, Stefan Hajdu, Harald K. Widhalm

**Affiliations:** 1 Clinical Division of Traumatology, Department of Orthopedics and Traumatology, Medical University of Vienna, Vienna, Austria; 2 Clinical Division of Orthopedics, Department of Orthopedics and Traumatology, Medical University of Vienna, Vienna, Austria; 3 Intensive Care Unit, Whittington Hospital, London, UK

**Keywords:** Coronavirus, Hip fracture, Suicide, Traumatology

## Abstract

**Objective:**

The outbreak of severe acute respiratory syndrome coronavirus 2 (SARS-Cov-2) and its associated illness, coronavirus disease 2019 (COVID-19), has led to a global health crisis burdening frontline emergency departments, including orthopedic and trauma units. The aim of this study was to provide an overview of the impact of the lockdown secondary to the pandemic on patient numbers and pattern of injuries at the department of traumatology of the Medical University of Vienna.

**Methods:**

This retrospective, descriptive study identified all patients admitted and enrolled onto the trauma registry at a level I trauma center, between 15 March 2020 and 30 April 2020 (lockdown) and compared them to those between 15 March 2019 and 30 April 2019 (baseline). Variables collected included patient age, sex, reason for hospital admission, place of injury, death, injury severity score (ISS), as well as American Society of Anaesthesiologists (ASA) score.

**Results:**

A total of 10,938 patient visits to the trauma emergency department were analyzed, 8353 presentations during the baseline period and 2585 during lockdown. Only 1869 acutely injured and 716 follow-up patients presented during lockdown, compared to 6178 and 2175, respectively, during baseline. Throughout the COVID-19 lockdown there were significant reductions in both workplace and traffic accidents, sports injuries, number of hospitalized patients, and overall visits to the trauma emergency department; however, the number of major traumas and hip fractures remained similar. Furthermore, there was a significant increase in the frequency of injuries at home as well as hospital admissions due to attempted suicide.

**Conclusion:**

Despite the reduction in total number of patients, trauma departments should continue to provide adequate service during lockdown considering that severe injuries showed no change. Conditions such as breakdown of social networks and limited access to mental health care and support might account for the significant rise in hospital admissions due to suicides. We recommend that more attention and effort should be made to prevent this excess of suicide deaths.

## Introduction

Coronavirus disease 2019 (COVID-19) has been declared a pandemic by the World Health Organization (WHO) with confirmed cases surpassing 4,179,479 and more than 287,034 estimated deaths across over 160 countries [1]. On 31 December 2019, 27 cases of pneumonia of unknown etiology were reported in the city of Wuhan [2], Hubei province, China. The causative agent was identified from throat swab samples performed at Chinese Centre for Disease Control and Prevention (CDC) on 7 January 2020 [2]. The virus was subsequently named severe acute respiratory syndrome coronavirus 2 (SARS-CoV-2), while the associated disease was called COVID-19 by the WHO.

The first PCR-positive case in Austria, as well as Switzerland, was reported on 05/02/2020 [3]; however, in the preceding weeks several suspected cases tested negative, giving the local authorities a false sense of control over the spread of the virus. Soon after, it was reported that popular skiing resorts throughout the Austrian Alps were already serving as transmission hubs for SARS-CoV‑2 [4]. It took several more days before the authorities recognized the impending threat to public health and reacted accordingly. As the rise in infections accelerated, a lockdown was declared on 14/03/2020 with full restrictions enforced from 16/03/2020 [5].

We hypothesize that the COVID-19 related lockdown due to the so-called first wave of the pandemic lead to reduced patient numbers and admissions, but a proportionally higher incidence of major trauma. The aim of the study was to provide an overview of the workflow at a level I trauma department during the 2020 lockdown and to assess its impact on patient numbers and injury pattern as compared to the corresponding period in 2019. This will identify areas for improvement and help establish guidelines in case of future waves/lockdowns [6].

## Patients and methods

### Patient selection

This retrospective, descriptive study identified all inpatient and outpatient visits to the trauma emergency department of a level 1 trauma center, during COVID-19 lockdown (15/03/2020–30/04/2020) and the corresponding baseline period (15/03/2019–30/04/2019). Patients had to fulfil both inclusion criteria: 1) presenting to any trauma emergency service, 2) presenting during either the lockdown or baseline timeframe. No exclusion criteria were defined. Separate visits by the same patient were considered distinct episodes. Data were collected from the clinical data registry with patients grouped based on demographics and predetermined subgroups.

### In-house procedures related to the pandemic

The Medical University of Vienna includes one of the biggest hospitals in Europe serving more than 2,000,000 inhabitants.

**Fig. 1:**
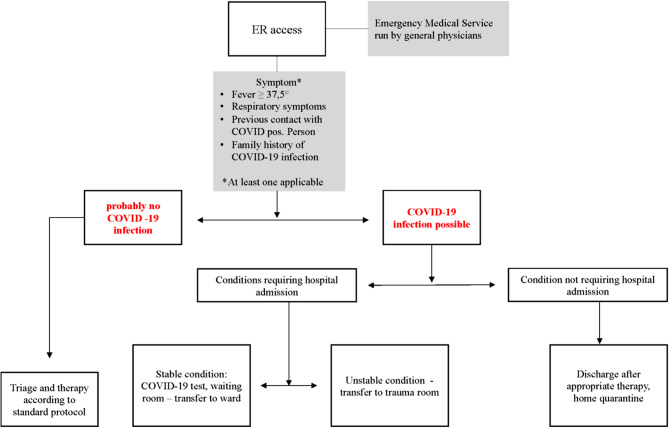
Algorithm of triaging patients at the department of traumatology

The hospital implemented several restrictions to access including body temperature screening and assessing for flu-like symptoms [7, 8]. Only patients with an appointment within a specific time slot were granted access to the hospital [9]. The trauma department imposed social distancing rules, ensuring seats were at least 1 m away from each other, and both staff and patients had to wear masks, when social distancing could not be adhered to (Fig. 1).

The availability of beds in the trauma department was significantly reduced, with areas being reassigned as designated COVID-19 beds. All nonemergency procedures, such as elective surgeries and noncritical clinical office activities were postponed or cancelled [10, 11]. If a surgery had to be performed before the laboratory and radiographic results were obtained, the patient was considered SARS-CoV‑2 positive, until the PCR test results were confirmed. Patients were screened with a nasopharyngeal swab PCR test at admission and then isolated in the rooms [12]. If a patient tested positive, the entire trauma team was then tested too. All trauma patients requiring urgent hospitalization were admitted into double rooms, with dedicated healthcare personnel and no external visitors thus limiting unnecessary exposure and risk.

All medical staff were instructed to wear full personal protective equipment (PPE), including FFP 2 medical masks, gowns, gloves, and eye protection (goggles or face shield) [13], during any clinical encounter. Instructional videos and teaching sessions were used to ensure correct donning and doffing of PPE and educating staff to minimize wasting limited resources.

Administrative personnel activity was reduced to a minimum and, where possible, remote working was implemented. By dividing the trauma department staff into 2 teams, alternating every 2 weeks, the time spent in the hospital was minimized while optimizing clinical efficiency.

Implementing these guidelines in the trauma department, as per the recommendation of the Medical University hospital, helped identify a total of 6 SARS-CoV‑2 positive patients, who were transferred to the COVID-19 dedicated clinic or ward. Furthermore, only one member of staff had to self-quarantine due to exposure at the hospital.

### Ethics statement

The study protocol was approved by the ethical committee (ethics commission Nr. 1517/2020).

### Statistical analysis

The numbers of patients presenting to the trauma department of the Medical University of Vienna during the lockdown period (15/03/2020–30/04/2020) were compared to the baseline period (15/03/2019–30/04/2019). Considering the events in each timeframe are independent of each other, the Poisson distribution is the most appropriate model.

Given the exact method is the most common way of calculating confidence intervals for small sample sizes, the following equation was used to determine the 95% confidence interval for each event ‘x’: χ^2^ (x^2^)

$$(\mathrm{x}^{2}(\upalpha /2,2*\mathrm{x})/2,\mathrm{x}^{2}(1-\upalpha /2,2*(\mathrm{x}+1))/2)$$

The *P*-value from the χ^2^-test for independence determined whether there was a statistically significant correlation between the different variables. Statistics were performed with the open-source software Python (version 3.4, Python Software Foundation, Wilmington, Delaware, USA).

## Results

### Overall patient numbers

Compared to baseline, there was a 69.75% reduction in both patients presenting to the trauma department and 61.49% reduction in admissions. No significant differences were identified in age and sex between the two timeframes (Table 1).

**Table 1:** Overall numbers of patients

Subgroups	Baseline	Lockdown	*p*
Total Patients (*n*)	9254	2939	0.001
**Presentations to the trauma emergency department (*n*, %)**	6178 (66.8%)	1869 (63.6%)	0.001
Male (*n*,%)	3299 (53.4%)	968 (51.8%)	0.01
Female (*n*,%)	2879 (46.6%)	901 (48.2%)	0.03
Age (mean, years)	38.0 ± 25.2	43.6 ± 23.6	0.3
Male (mean ± SD, years)	33.5 ± 36.6	39.7 ± 34.5	0.4
Female (mean ± SD, years)	43.1 ± 23.4	47.7 ± 20.1	0.3
**Follow-up patients (*n*,%)**	2175 (23.5%)	716 (24.4%)	0.001
Male (%)	1174 (54%)	398 (55.6%)	0.03
Female (%)	1001 (46%)	318 (44.4%)	0.02
Age (mean ± SD, years)	39.2 ± 23.5	42.7 ± 21.5	0.1
Male (mean ± SD, years)	31.2 ± 33.4	34.7 ± 31.9	0.2
Female (mean ± SD, years)	40.3 ± 22.4	40.1 ± 27.8	0.1
**Hospitalized patients (*n*,%)**	901 (9.7%)	347 (11.8%)	0.001
Male (%)	442 (49.1%)	175 (50.4%)	0.1
Female (%)	458 (50.9%)	172 (49.6%)	0.2
Age (mean ± SD, years)	62.1 ± 10.4	63.9 ± 12.3	0.1
Male (mean ± SD, years)	54 ± 23.4	57.8 ± 20.5	0.2
Female (mean ± SD, years)	70 ± 15.4	70.1 ± 13.2	0.3

### Major trauma and polytrauma patients

Although major traumas, defined as an injury severity score (ISS) greater than 15, reduced from 50 to 43 during lockdown, this was not statistically significant (*p* = 0.2). Of the 43 major trauma cases admitted during lockdown, 22 (51.2%) were mainly do it yourself (DIY)-related falls, 6 (14.0%) traffic-related, 10 (23.3%) suicide attempts, 4 (9.3%) outdoor injuries, and only 1 (2.3%) was crime-related. Although DIY-related injuries remained the most common major trauma presentation, there was a significant reduction during lockdown (22 vs. 17 respectively, *p* = 0.003). Admissions to the trauma room due to suicide increased 10-fold during lockdown (10 vs. 1, *p* = 0.003) while traffic and outdoor-related injuries were more than halved (6 vs. 13, *p* = 0.01 and 4 vs. 15, *p* = 0.04, respectively).

**Fig. 2:**
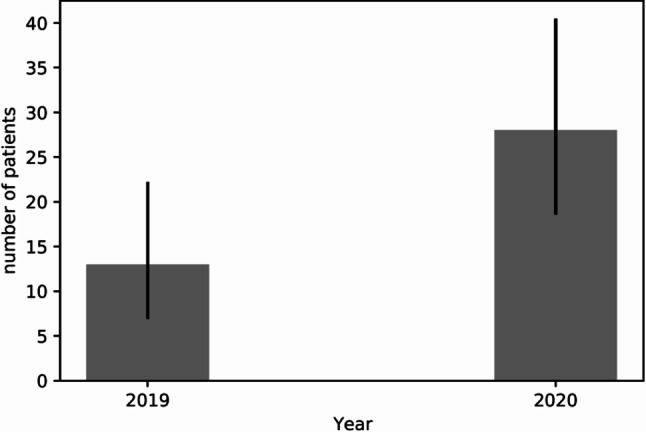
Accidents at home

The severity of injuries among all major trauma patients significantly increased during lockdown (16.05 ± 17.09 vs. 11.98 ± 729, *p* = 0.004). Furthermore, mortality increased to 14% (*n* = 6), with the average ISS among these patients being 45 ± 23.24. Most major traumas occurred at home during lockdown (*n* = 28, 65.1%) (Fig. 2), almost double from baseline (*n* = 13, 26%) when most major traumas occurred on the street (*n* = 32, 64%). There was a trend towards an increase in the age of major trauma patients during lockdown (45.2 years SD 26.9 vs. 42.9 years SD 27.3, *p* = 0.2); however, neither age nor the male:female ratio were statistically significant. Notably, time to surgery was significantly prolonged by 45% (median ± interquartile range, IQR; 144 min + 258.5 vs. 157 min + 62.3 *p* = 0.001) during lockdown (Table 2).

**Table 2:** Characteristics of major trauma admissions

Patients	Baseline	Lockdown	*p*
**Trauma Room Patients**			
**Total (*n*)**	50	43	0.2
Male (*n*,%)	37 (74%)	30 (69.8%)	0.1
Female (*n*,%)	13 (26%)	13 (30.2%)	0.1
Age (mean, years)	42.88 ± 27.31	45.19 ± 26.87	0.2
Male (mean ± SD, years)	40.27 ± 24.2	46.17 ± 24.91	0.2
Female (mean ± SD, years)	52.33 ± 35.48	42.92 ± 31.94	0.2
Injury severity score (ISS)	11.98 ± 7.29	16.05 ± 17.09	0.004
**Location of trauma**			
Home (*n*,%)	13 (26%)	28 (65.1%)	0.004
Street (*n*,%)	32 (64%)	14 (32.6%)	0.003
Work (*n*,%)	5 (10%)	1 (2.3%)	0.003
**Reason of trauma (*n*, %)**			
Fall (*n*,%)	17 (34%)	22 (51.2%)	0.003
Traffic (*n*,%)	13 (26%)	6 (14%)	0.01
Suicide (*n*,%)	1 (2%)	10 (23.3%)	0.003
Crime (*n*,%)	4 (8%)	1 (2.2%)	0.002
Outdoor (*n*,%)	15 (30%)	4 (9.3%)	0.04
**Time until surgery (min)**	157 + 62.3* (maximum time until surgery: 219.3 min)	221 + 258.5* (maximum time until surgery: 479.5 min)	0.001
**Death (*n*,%)**	2 (4%)	6 (14%)	0.001
**Intensive care (*n*,%)**	13 (26%)	10 (23.3%)	0.1

### Suicide and domestic violence

**Fig. 3:**
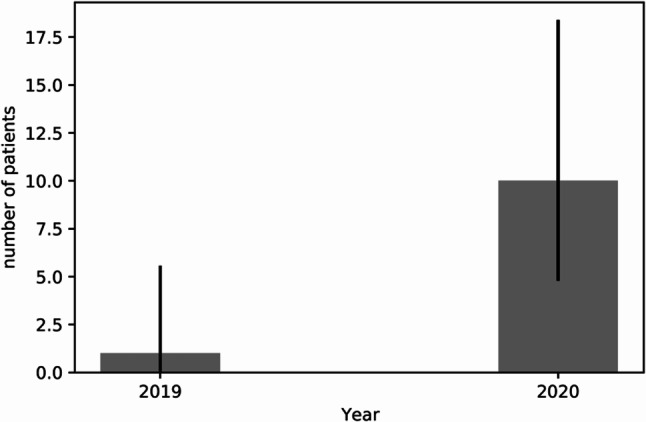
Suicide cases—comparison of incidence during lockdown and baseline

During lockdown, patients presenting due to suicide attempts significantly increased (10 vs. 5; 0.003), especially within the male subgroup (8 vs. 2, *p* = 0.001) (Fig. 3). Domestic violence, however, decreased significantly in terms of total cases (18 vs. 38; *p* = 0.003) as well as among the female cohort (16 vs. 33, *p* = 0.005) but the male-to-female ratio remained similar (Table 3).

**Table 3:** Overview of suicide and domestic violent cases

Subgroup	Baseline	Lockdown	*p*
**Suicide**			
Total (*n*)	5	10	0.003
Age (mean ± SD, years)	34 ± 7.6	39 ± 5.4	0.02
Male (*n*,%)	2 (40%)	8 (80%)	0.001
Female (*n*,%)	3 (60%)	2 (20%)	0.02
**Domestic violence**			
Total (*n*)	38	18	0.003
Age (mean ± SD, years)	31 ± 10.5	32 ± 15.4	0.01
Male (*n*,%)	5 (13.2%)	2 (11.1)	0.2
Female (*n*,%)	33 (86.8%)	16 (89.9)	0.005

### Surgery

There was a 69.4% reduction in surgeries (497 vs. 152, *p* = 0.001) carried out during lockdown, mostly due to the lack of elective surgeries such as arthroscopy (76 vs. 4, *p* = 0.001) and revision surgery (56 vs. 7, *p* = 0.001). (Table 4).

**Table 4:** Overview of all surgeries

Surgeries	Baseline	Lockdown	*p*
**Surgeries performed**			
Total (*n*)	497	152	0.001
**Acute surgical cases**			
Wounds (*n*, %)	85 (17.1%)	18 (11.8%)	0.003
Tendons, nerves, vessels (*n*, %)	39 (7.8%)	16 (10.5%)	0.02
Osteosynthesis (*n*, %)	177 (35.6%)	70 (46.1%)	0.03
Hip fractures (*n*, %)	64 (12.9%)	37 (24.3%)	0.03
**Elective surgical cases**			
Arthroscopy (*n*, %)	76 (15.3%)	4 (2.6%)	0.001
Revision surgeries (*n*, %)	56 (11.3%)	7 (4.6%)	0.001

### Hospitalization

Although there was a significant reduction in the absolute number of concussions (23 vs. 114, *p* = 0.03) and hip fractures (37 vs. 64, *p* = 0.001), they remained the two most common injuries requiring admission during lockdown. (Table 5).

**Table 5:** The two most common diagnosis requiring admission in the trauma department

Diagnosis at admission	Baseline	Lockdown	*p*
Total admissions (*n*)	901	347	0.002
**Concussion**			
Total (*n*,%)	114 (12.7%)	23 (6.6%)	0.03
Male (*n*,%)	57 (50%)	17 (73.9%)	0.01
Age (mean ± SD, years)	64.7 ± 24.5	66.9 ± 6.9	0.4
Female (*n*,%)	57 (50%)	6 (26.2%)	0.01
Age (mean ± SD years)	63.4 ± 8.4	65.9 ± 9.4	0.4
**Hip fractures**			
Total (*n*,%)	64 (7.1%)	37 (10.7%)	0.001
Male (*n*,%)	8 (13.2%)	4 (11.1%)	0.2
Age (mean ± SD, years)	79.8 ± 11.5	80.51 ± 12.3	0.3
Female (*n*,%)	56 (86.8%)	33 (88.9%)	0.001
Age (mean ± SD years)	81.33 ± 11.03	81.68 ± 9.72	0.2

**Fig. 4:**
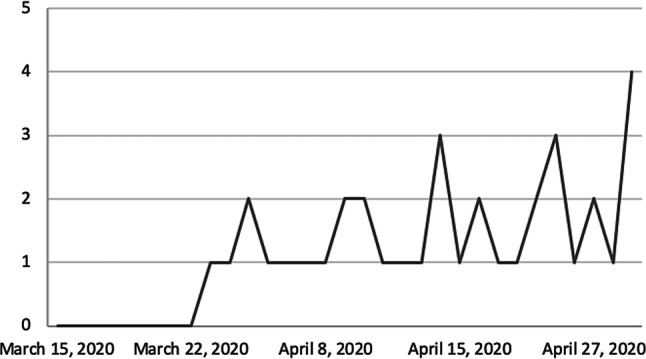
Numbers of hip fractures per day admitted to the trauma department

Hip fractures during lockdown trended towards a higher proportion of total injuries requiring admission (10.7% vs. 7.1%). Furthermore, following the first 7 days of lockdown with no new hip fracture admissions, there was an upward daily trend (Fig. 4).

Except for a statistically significant reduction in meniscus lesions (92 vs. 14, *p* = 0.001) and ankle sprains (420 vs. 81, *p* = 0.003) there were no other prominent changes within sports-related injuries. (Table 6).

**Table 6:** Sports-related injuries requiring admission

Sports-related Injury	Baseline	Lockdown	*p*
**Ankle sprains**			
Total (*n*)	420	81	0.005
Age (mean ± SD, years)	30.8 ± 16.7	36.3 ± 18.7	0.2
Male (*n*,%)	220 (52.4%)	41 (50.6%)	0.2
Female (*n*,%)	200 (47.6%)	40 (49.4%)	0.4
**Meniscus lesions**			
Total (*n*)	92	14	0.001
Age (mean ± SD, years)	38 ± 17.2	47 ± 20.1	0.004
Male (*n*,%)	54 (58.7%)	5 (35.7%)	0.002
Female (*n*,%)	38 (41.3%)	9 (64.3%)	0.01
**Knee contusion**			
Total (*n*)	459	163	0.005
Age (mean ± SD, years )	50 ± 28	53.7 ± 26.9	0.1
Male (*n*,%)	225 (49.0%)	87 (53.4%)	0.1
Female (*n*,%)	234 (51.0%)	76 (46.6%)	0.2

Of the patients 6 (1.7%) were tested positive for COVID-19 during lockdown and were transferred to a COVID-19 dedicated hospital.

## Discussion

During the national lockdown there was a significant reduction in the overall patient volume inward and outward but, unlike Christey et al. [14] there was no significant reduction in major trauma; however, the higher ISS among these trauma cases, along with further delays to surgery caused by SARS-CoV‑2 PCR testing, may explain the increase in death rate from 4% to 14% during lockdown. Although there was no decrease in major injuries, the place of injury significantly shifted, as expected, from outdoors to at home.

Of particular note, this study identified a significant increase in admissions due to attempted suicide. This highlights that the impact of COVID-19 on mental health may be more profound than anticipated, in line with suggestions that suicide rates will rise as the pandemic continues [15]. Given the long-term effects on the general population, the economy, as well as the vulnerable groups, suicide prevention will need urgent consideration [16]. Data from previous pandemics suggest increasing rates of suicide, as seen in the USA during the 1918–1919 [16] influenza pandemic and among the older population of Hong Kong during the 2003 SARS epidemic [17, 18]. Therefore, we hypothesize that mental health consequences are likely to be present for longer and will peak later than the pandemic.

The number of hospital admissions due to domestic violence during lockdown significantly reduced by 40% by the end of April. This is not supported by the literature, with China reporting a tripling of domestic violence during lockdown [19]. Furthermore, in Europe, France showed a 30% increase in domestic violence [20] and Spain reported rising rates of domestic violence-related homicide [21]. Considering the social breakdown occurring during both lockdown and natural disasters, there is evidence suggesting that domestic violence rates increase, and remain higher than usual, for several months after the catastrophic event [22]. Furthermore, the number of unreported cases of domestic violence remains debated with the literature suggesting only between 2.5–15% of cases being reported [23]. Thus, it is critical that governments across the world enable services, including therapists and helpline practitioners working in the field of domestic and sexual violence, to stay open [19].

Although there was a 42% reduction in hip fractures admitted to hospital (*n* = 64 vs. *n* = 37; *p* = 0.001), they represented a higher proportion of the total admissions during lockdown (0.6% vs. 2%; *p* = 0.001). In the first 7 days of the lockdown there were no new hip fracture cases presented to the trauma emergency department (Fig. 4). This suggests that the reduction in cases was most likely influenced by the beginning of lockdown. Kenanidis et al. reported low numbers of hip fractures during the early phase of the Greek lockdown with a significantly upward trend towards end of lockdown. The authors speculated that the impact on lifestyle and psychology of the older citizens during the early phase of the lockdown was critical. Initially the limited mobility around the house reduced the number of falls and osteoporotic hip fractures; however, by the end of lockdown, the increase in the incidence of hip fractures probably relates to acclimatizing to the restrictive measures and realizing the controlled spread of the virus across the country coaxed reduced adherence to the lockdown [24]. Our results support this theory, with the number of patients with hip fractures dropping to zero during the early phase and rising by the end of lockdown.

Delays in the treatment of hip fractures due to secondary effects of the COVID-19 pandemic, such as hospital reorganization need to be analyzed in further studies. These delays could affect hip fracture patients, as delays in operative treatment are well known to increase mortality [25].

It is clear from the literature and our results that even during lockdown, a certain number of trauma cases will continue to present to hospital. Although the reduction in cases is unsurprising, considering the emptier streets and self-isolating initiatives led by government policy, the question remains: where are all the minor injuries? Are they being self-treated at home? One theory suggests that the sudden surge in COVID-19 cases, along with the mediatization of the global emergency, led to a widespread sense of overstretched healthcare systems [26]. Furthermore, patients may well have avoided hospitals as they feared they would catch the virus and fall ill. Considering these circumstances it is possible that a reduction in triggers resulting from isolation will be followed by a surge as we emerge from isolation and return to work and other activities. Thus, trauma may not have been prevented as much as deferred [27].

Physical activity plays a crucial role in public health—even with a global pandemic and lockdown in place, physical activity was not only permitted, but encouraged. Medical universities recommended moderate types of exercise, from jogging and Nordic walking to brisk walks in the park and forest trails [28] and 80% of jogging injuries are due to overstraining and cannot be associated with acute accidents [29]. During lockdown there was a significant reduction in both group-related and individual-related injuries. One explanation is that the public was mindful of the consequences of moderate to severe injuries and thus reduced their daily activities to avoid injuries. Another possibility is that most injuries associated with solo activities do not require medical intervention but rather need to be rested, something people could try at home. Only the injuries which persisted through the rest period ended up presenting to the trauma department, generating a self-selection bias during lockdown.

## Limitations of the study

This study does have limitations. There may have been other confounding factors, such as weather conditions, that may have affected trauma numbers between years that we have not accounted for, and data collection was only from one center, which could differ from all other Austrian trauma center data.

## Conclusion

Although trauma of all age groups and severities will continue to occur, the tendency during a lockdown will be a greatly reduced case load. Nevertheless, with no significant drop in major injuries, resources need to remain readily available for any future waves. We propose that trauma departments should continue to review and update safety plans and guidelines in order to provide full services until any significant resource restriction occurs. Furthermore, this crisis has provided important lessons for staff organization and leadership, including efficient management of surgeries and trauma with a reduced but dedicated staff. The importance of versatility in managing limited resources has been highlighted, always adapting to an ever-changing situation. This will ensure the highest levels of service are maintained, reducing complications and ultimately improving patient outcomes.
